# High-Throughput Sequencing Facilitates Characterization of a “Forgotten” Plant Virus: The Case of a Henbane Mosaic Virus Infecting Tomato

**DOI:** 10.3389/fmicb.2018.02739

**Published:** 2018-11-19

**Authors:** Anja Pecman, Denis Kutnjak, Nataša Mehle, Magda Tušek Žnidarič, Ion Gutiérrez-Aguirre, Patricija Pirnat, Ian Adams, Neil Boonham, Maja Ravnikar

**Affiliations:** ^1^Department of Biotechnology and Systems Biology, National Institute of Biology, Ljubljana, Slovenia; ^2^Jožef Stefan International Postgraduate School, Ljubljana, Slovenia; ^3^KZ Agraria Koper z.o.o., Koper, Slovenia; ^4^Fera Science Ltd., York, United Kingdom; ^5^Institute for Agri-Food Research and Innovation, Newcastle University, Newcastle upon Tyne, United Kingdom; ^6^Wine Research Centre, University of Nova Gorica, Nova Gorica, Slovenia

**Keywords:** potyvirus, henbane mosaic virus, tomato, high-throughput sequencing, host range analysis, phylogeny

## Abstract

High-throughput sequencing has dramatically broadened the possibilities for plant virus research and diagnostics, enabling discovery of new or obscure viruses, and virus strains and rapid sequencing of their genomes. In this research, we employed high-throughput sequencing to discover a new virus infecting tomato, *Henbane mosaic virus (Potyvirus*, *Potyviridae*), which was first discovered at the beginning of 20th century in the United Kingdom in cultivated henbane. A field tomato plant with severe necrotic symptoms of unknown etiology was sampled in Slovenia and high-throughput sequencing analysis using small RNA and ribosomal RNA depleted total RNA approaches revealed a mixed infection with *Potato virus M* (*Carlavirus*, *Betaflexiviridae*), *Southern tomato virus* (*Amalgavirus*, *Amalgamaviridae*) and henbane mosaic virus in the sample. The complete genomic sequence of henbane mosaic virus was assembled from the sequencing reads. By re-inoculation of the infected material on selected test plants, henbane mosaic virus was isolated and a host range analysis was performed, demonstrating the virus was pathogenic on several plant species. Due to limited metadata in public repositories, the taxonomic identification of the virus isolate was initially putative. Thus, in the next step, we used small RNA sequencing to determine genomic sequences of four historic isolates of the virus, obtained from different virus collections. Phylogenetic analyses performed using this new sequence information enabled us to taxonomically position *Henbane mosaic virus* as a member of the *Potyvirus* genus within the chili veinal mottle virus phylogenetic cluster and define the relationship of the new tomato isolate with the historic ones, indicating the existence of at least four putative strains of the virus. The first detection of henbane mosaic virus in tomato and demonstration of its pathogenicity on this host is important for plant protection and commercial tomato production. Since the virus was initially present in a mixed infection, and its whole genome was not sequenced, it has probably been overlooked in routine diagnostics. This study confirms the applicability of a combination of high-throughput sequencing and classic plant virus characterization methods for identification and phylogenetic classification of obscure viruses and historical viral isolates, for which no or limited genome sequence data is available.

## Introduction

The immense sequence data generating potential of high-throughput sequencing (HTS) has enabled an accelerated discovery of new virus species in recent years (Barba et al., 2013; Massart et al., 2014; Adams and Fox, 2016) and increased resolution of viral population and evolution studies (Kutnjak et al., 2017). However, currently there is a gap between sequence discovery and biological characterization of new viral species, the latter requiring time-consuming research efforts (Massart et al., 2017). In the present research we address another angle of this problem; we used HTS to detect and sequence the genome of a known plant virus, namely henbane mosaic virus (HMV, genus *Potyvirus*, family *Potyviridae*), which had a poorly characterized genome, despite being the subject of several biological studies in the past.

HMV was discovered in cultivated henbane (*Hyoscyamus niger*) in 1932 in the United Kingdom (Rothamsted Experimental Station) (Hamilton, 1932) and later reported as HMV-R (Rothamsted) (Kitajima and Lovisolo, 1972). However, due to difficulties characterizing viruses in the pre-molecular era, new isolates might sometimes be named differently, as a new virus, based on the induced symptoms and hosts, thus the following historical overview could be incomplete. The virus was later reported in the United Kingdom in *Atropa belladonna* (Smith, 1945) and in *Datura stramonium* (Bradley, 1952). HMV was then reported in 1970 in Italy, infecting *Datura inermis*, *D. stramonium*, and *Physalis alkekengi*. *P. alkekengi* was considered the main host and consequently the new strain was labeled as HMV-A (Lovisolo and Bartels, 1970). The virus was found also in Hungary (Horváth et al., 1988) in *D. stramonium* and designated as HeMV-W/H. Additionally, there were reports of the virus from Germany and India, where henbane is grown for medical purposes (stated in Lovisolo, 1992). The suggested main host plants for HMV are different species from the Solanaceae family (Govier and Plumb, 1972). In nature HMV can be transmitted by aphids (Hamilton, 1932; Bradley, 1952; Lovisolo and Bartels, 1970; Govier and Plumb, 1972) and by mechanical inoculation in greenhouse plants (Govier and Plumb, 1972). Its particles are filamentous in shape (Govier and Plumb, 1972) with a length, estimated by electron microscopy to be approximately 850 nm (Lovisolo and Bartels, 1970).

In all the previous reported cases HMV was found in different solanaceous plants, which were not crop or vegetable plants, important from the agricultural perspective. However, in this study, we report for the first time natural HMV infection in field-grown tomato, found in Slovenia in 2015 (Ankaran) showing severe necrotic symptoms. Tomato (*Solanum lycopersicum* L.) is one of the most widely grown vegetable crops worldwide (FAOSTAT, 2001). Tomato is a natural host for many plant viruses (Brunt et al., 1996; Mihara et al., 2016) which cause significant economic losses by reducing crop quality and quantity, thus timely detection of emerging viral diseases in tomato is crucially important (Hanssen et al., 2010). HMV was detected in a symptomatic tomato plant, using HTS, in a mixed infection with *Potato virus M* (*Carlavirus, Betaflexiviridae*) and *Southern tomato virus* (*Amalgavirus*, *Amalgamaviridae*). Prior to this study only a short fragment of the HMV genome sequence was available in databases with very limited metadata, thus, the initial species identification was only putative. To address this gap, we have obtained four other historic isolates of the virus from two different virus collections and used small RNA high-throughput sequencing to obtain complete genome sequences. Besides the first detection of the HMV in an important vegetable crop (tomato), we also report the first detection of the virus in Slovenia and the first complete genome sequence. Moreover, we demonstrate the utility of HTS for the rapid characterization of known plant virus species with no or little sequence information. We discuss implications of the approach for other viral species and discuss the power of the method for the revision of historical virus isolates and viral collections in general.

## Materials and Methods

### Description of Isolates Included in the Analysis

A tomato (*S. lycopersicum* L.) sample (with laboratory diagnostic identification number D159/15) with necrotic disease symptoms was collected in Slovenia (Ankaran) in the summer of 2015 and stored at -80°C for further analyses. The sample contained a mixed infection with HMV, PVM, and STV. The HMV virus isolate found in this sample will be designated throughout the manuscript as isolate HMV-SI/L representing henbane mosaic virus (HMV) from Slovenia (SI) from *S. lycopersicum* (L).

Four other isolates were sourced from virus collections as lyophilized leaf material and stored at -20°C for further analyses. Isolates HMV-146 and HMV-R were obtained from the Institute for Sustainable Plant Protection (IPSP), Italy. The original host of isolate HMV-146 is *D. inermis* from Torino, Italy, and the origin of isolate HMV-R is Rothamsted, United Kingdom. Isolates henbane mosaic virus ATCC^®^ PV-76^TM^ and henbane mosaic virus ATCC^®^ PV-79^TM^ were purchased from the American Type Culture Collection (ATCC) and will be from this point on labeled as HMV-PV-76 and HMV-PV-79, respectively. The original host of isolates HMV-PV-76 and HMV-PV-79 is *H. niger* (henbane), and their origin is United States (California) and England, respectively. Detailed information for the isolates is shown in Supplementary Table 1.

### First Test on Sample HMV-SI/L

Tomato sample from Slovenia was inspected using transmission electron microscopy and tested by ELISA for Impatiens necrotic spot virus (INSV, genus *Orthotospovirus*, family *Tospoviridae*), tomato spotted wilt virus (TSWV, genus *Orthotospovirus*, family *Tospoviridae*), potato virus S (PVS, genus *Carlavirus*, family *Betaflexiviridae*) and potato virus M (PVM, genus *Carlavirus*, family *Betaflexiviridae*). ELISA was performed using kits containing virus specific antibodies as follows: INSV (Loewe Biochemica GmbH, Germany), TSWV (Adgen, United Kingdom), PVS (Bioreba AG, Switzerland), and PVM (Bioreba AG, Switzerland). Additionally, selected test plants (*S. lycopersicum* cv. Moneymaker, *Nicotiana rustica*, *Nicotiana tabacum* cv. White Burley, *Nicotiana benthamiana*, *Nicotiana clevelandii*, *Nicotiana glutinosa*, *Chenopodium quinoa*, *D. stramonium* and *Capsicum annuum*) were mechanically inoculated with a (1:10) dilution of the original sample in phosphate buffer (0.02 M with 2% PVP) and applied to the first two to three completely expanded leaves dusted with carborundum. Test plants were visually inspected for symptoms and tested by electron microscopy and ELISA for PVM 4 weeks after mechanical inoculation. The original plant sample and symptomatic test plants were also tested for STV using RT-PCR (Sabanadzovic et al., 2009).

### RNA Isolation and High-Throughput Sequencing

For all of the reverse-transcription PCRs (RT-PCRs) in the testing and confirmation steps, RNA was isolated from leaf samples using RNeasy Plant Mini Kit (Qiagen, Netherlands) following the manufacturer’s protocol, with some minor modifications as follows. RLT buffer without β-mercaptoethanol was added to plant material and RNA was eluted from the RNeasy Mini Spin columns using 50 μl of RNase-free warm water (65°C). All of the samples were stored at -80°C between sampling and extraction. Isolated RNA was stored at -80°C, when not in use.

Ribosomal RNA depleted total RNA sequencing was performed for the sample HMV-SI/L. The RNeasy Plant Mini Kit (Qiagen, Netherlands) was again used for RNA isolation, including the optional DNase treatment (RNA Cleanup protocol; RNeasy Mini Kit; Qiagen, Netherlands). Ribosomal RNA was then depleted from the total RNA and libraries for sequencing were prepared using the ScriptSeqTM Complete Kit (plant leaf) (Illumina, United States). The libraries were sequenced using MiSeq (Illumina, United States) in 2 × 300 bp (V3) mode.

Small (s)RNA sequencing was performed for samples HMV-SI/L; HMV-146; HMV-R; HMV-PV-76; HMV-PV-79. In this case, total RNA was isolated using TRIzol reagent (Invitrogen, United States) following the manufacturer’s protocols. Total RNA from the four samples was sent to Seqmatic LLC (United States) for sRNA library preparation and sequencing using a HiSeq 2000 (Illumina, United States) in 1 × 50 bp mode.

### Analysis of HTS Data for Virus Detection, Reconstruction of HMV Genomes and Their Annotation

In the first stage, HTS of sample HMV-SI/L was performed for both, rRNA depleted total RNA and sRNA. Two different detection pipelines were used to detect viral sequences in the HTS data for both approaches as previously described (Pecman et al., 2017). In both datasets, several contig sequences matched different potyviruses with relatively low similarity, indicating the presence of an unknown potyvirus (later identified as HMV). Since contigs assembled from sRNA reads using the above-cited pipeline were relatively short, we performed an additional assembly of sRNA reads using SPAdes (Bankevich et al., 2012; Nurk et al., 2013). The parameter “careful” and combined k-mer sets of 15, 17, 19, and 21 were applied to produce long assembled sequences (Barrero et al., 2017). For each of the two sequencing approaches, all of the contigs matching potyvirus sequences were further assembled to obtain a complete or near complete viral genome sequence using CLC Genomic Workbench 10 (Qiagen). The final consensus genomic sequences assembled from rRNA depleted total RNA and sRNA data were compared to confirm they were identical. Finally, the trimmed reads from both datasets were mapped to the final complete consensus viral genomic sequence. The mapping results were visually inspected for any errors. For genome assembly confirmation six pair of primers were designed (Supplementary Table 2). RT-PCR reactions were carried-out using those primers and OneStep RT-PCR Kit (Qiagen, Netherlands). The reaction conditions were 50°C for 30 min, 95°C for 15 min, followed by 35 cycles of 94°C for 30 s, 51°C for 60 s and 72°C for 60 s. The amplicons obtained by RT-PCR were purified using MinElute PCR Purification Kit (Qiagen, Netherlands) and submitted to Sanger sequencing (GATC Biotech AG, Germany). The sequences obtained were aligned against the HMV-SI/L genome to confirm that they were identical.

For samples HMV-146, HMV-R, HMV-PV-76 and HMV-PV-79, sRNA sequencing was performed and the resulting datasets were analyzed to identify viral sequences using the pipeline described previously (Pecman et al., 2017). Complete consensus genomic sequences of HMV in these samples were reconstructed by assembly of sRNA reads as described above. Finally, the sRNA reads derived from each isolate were mapped to the corresponding reconstructed whole genome consensus sequences and the mapping results were visually inspected for any errors.

Furthermore, in order to validate the assemblies within the repeated region (TATATA) around position 9980 nt, we designed universal PCR primers for all HMV isolates: HMV-UNI-F: 5′-TTAGCCCGATATGCTTTC-3′ and HMV-UNI-R: 5′-CTATCTTCCACTTCAGGT-3′. The RT-PCR reaction was performed using OneStep RT-PCR Kit (Qiagen, Netherlands). The reaction conditions were 50°C for 30 min, 95°C for 15 min, followed by 35 cycles of 94°C for 30 s, 48°C for 60 s, and 72°C for 60 s. The amplicons obtained by RT-PCR were purified using MinElute PCR Purification Kit (Qiagen, Netherlands) prior to sequencing (GATC Biotech AG, Germany). The sequences obtained were aligned against the HMV genomes and the repeated region was validated.

The polyproteins of each of the HMV isolates were annotated using Sequin^1^ and the individual putative cleavage sites for each gene product were manually determined based on known cleavage sites for other potyviruses reported in the literature (King et al., 2012). The predictions of molecular weight for putative viral protein products were calculated using the Protein Molecular Weight Calculator^2^. Genomic sequences of HMV-SI/L; HMV-146; HMV-R; HMV-PV-76; HMV-PV-79 were deposited in GenBank under accessions numbers MH779472, MH779473, MH779474, MH779475, and MH779476, respectively (Supplementary Table 1).

### RT-PCR Assay for HMV-SI/L Detection

In order to confirm HMV-SI/L infection in test plants, one (HMV-NIb-F: 5′-GTCAAGAAGTTCAAAGGG-3′ and HMV-CP-R: 5′-TACACCACACCATCAATC-3′) out of the six primer pairs designed for genome assembly was used. Negative controls for RNA isolation and no template controls were also tested. RT-PCR was done as described above using the OneStep RT-PCR Kit (Qiagen, Netherlands) in a 10 μl reaction volume.

### Pairwise Comparisons of Viral Genome Sequences and Phylogenetic Analyses

Pairwise comparison between nucleotide sequences of HMV isolates was performed in CLC Genomic Workbench 11. The comparison was done firstly by comparing whole genome sequences for the five isolates sequenced (HMV-SI/L; HMV-146; HMV-R; HMV-PV-76; HMV-PV-79). The comparison was also performed using partial genome sequences (1600 nt) to enable comparison with the published sequence of isolate HMV PHYS/H-Hungary (accession number AM184113). The results were visualized as heatmaps. In addition, nucleotide identities of the HMV-SI/L polyprotein sequence were compared with four other isolates (HMV-146, HMV-R, HMV-PV-76, and HMV-PV-79) and visualized using SimPlot 3.5.1 (Lole et al., 1999). A plot of nucleotide identity was obtained using the polyprotein sequence of isolate HMV-SI/L as a query sequence; HMV-R, HMV-PV-76 and HMV-PV-79 were compared to the query sequence as a group, since they were highly similar to each other (above 99% of nucleotide identity). A sliding window of 400 nt was used across the alignment in steps of 40 nt.

To reveal phylogenetic relationships between henbane mosaic virus isolates and other members of *Potyviridae* family we performed a phylogenetic analysis including complete viral polyprotein sequences of the five HMV isolates from this study and other members of the *Potyviridae* family. Alignment of the complete polyproteins of the known members of *Potyviridae* family was obtained from the International Committee on Taxonomy of Viruses (ICTV) resources^3^, including 130 viral species. Sequences of the five HMV isolates sequenced in this study were added to the alignment. Additionally, we performed blastn searches using HMV-SI/L against the NCBI *nt* database and added also two viral species with sequenced complete genomes (KY623506 and MF997470) with relatively high similarity to HMV (detected within first 100 blastn hits), which were not present in the initial ICTV alignment. All sequences were codon aligned applying MUSCLE in MEGA7 (Tamura et al., 2013). Phylogenetic trees were constructed from the alignment obtained in MEGA7 using a maximum likelihood algorithm and applying the GTR + G + I substitution model, which was determined to best fit the data. Bootstrap replication (100 pseudoreplicates) was used to assess the statistical support of the groups on the tree.

We aligned also partial genome sequences (∼1600 nt) of the six HMV isolates (HMV-SI/L, HMV-146, HMV-R, HMV-PV-76, HMV-PV-79, and Hungarian HMV PHYS/H) and other *Potyvirus* species, which clustered in the same group as HMV isolates in the above described complete polyprotein analysis. Additionally, we performed blastn searches of the partial genome sequence of HMV-SI/L against the NCBI *nt* database and added one viral species with partial genome sequence (FJ543110) and with relatively high similarity to HMV (detected within first 100 blastn hits), which was not present in the initial ICTV alignment. Several *Rymovirus* species (Y09854, AY623626, AY623627) were used as an outgroup. As described above, the partial genome sequences were codon aligned applying MUSCLE in MEGA7 and phylogenetic tree was constructed in MEGA7 on the basis of this alignment using maximum likelihood algorithm with GTR + G + I substitution model. Bootstrap replication (1000 pseudoreplicates) were used to assess the statistical support of the groups within the tree.

### Isolation of HMV-SI/L From Mixed Infection and Analysis of Its Host Range

HMV was detected in the field-grown tomato sample in a mixed infection with two other viruses. The isolation of HMV from the mixed infection was achieved by several re-inoculation steps. A tomato sample, positive for HMV, PVM, and STV, was used for inoculation of several test plants (see section “First Test on Sample HMV-SI/L”). The *N. clevelandii* plant material, to which PVM and HMV were successfully transferred, was then used to inoculate several test plants of *S. lycopersicum* cv. Moneymaker. The leaf material of *S. lycopersicum* with a single infection of HMV (confirmed by ELISA and RT-PCR) was collected and further used as an inoculum for the host range analysis.

A total of 20 different plant species/varieties from several botanical families were selected (Table 1) and included in the host range analysis. Since the virus was found in tomato, species from the Solanaceae family were mainly included in the analysis, however, to test for potential new hosts, plants from three other families were also included. Mechanical inoculation was performed as explained in section “First Test on Sample HMV-SI/L.” For each plant species, we mechanically inoculated 8 plants with infected material, and 4 plants were mock inoculated with buffer only, as a negative control. Inoculated test plants were grown in the quarantine greenhouse (20 ± 5°C; 16 h photoperiod) and the appearance of disease symptoms were recorded weekly. Three weeks post inoculation, locally and/or systemically infected leaves of symptomatic and asymptomatic plants were collected as pooled samples (separate pools were collected for each category: local leaves and systemic leaves, separately for symptomatic plants and asymptomatic plants). Collected pooled samples were tested for HMV with RT-PCR using the primers HMV-NIb-F and HMV-CP-R (Supplementary Table 2). In parallel, to confirm the absence of PVM, all samples were analyzed using reverse-transcription real-time PCR for PVM (Yang et al., 2014). Mock inoculated plants were analyzed in the same manner. The absence of STV was already confirmed in all of the diagnostic test plants used in first stage of the experiments (which were the source for further experiments).

**Table 1:** Symptoms of HMV-SI/L isolate developed on different test plants and their confirmation using RT-PCR.

Test plants		Number of symptomatic/ asymptomatic plants	Local symptoms on inoculated leaves	RT-PCR result inoculated symptomatic/ asymptomatic leaves	Symptoms on non-inoculated leaves	RT-PCR result non-inoculated symptomatic/ asymptomatic leaves


Botanical family	Species					
Solanaceae	*Solanum lycopersicum* Moneymaker	7/1	Leaf deformation, necrosis, chlorosis,	NA	Leaf deformation, blistering, necrosis, chlorosis,	+/-
	*Solanum lycopersicum* Rio grande	8/0	Leaf deformation, necrosis, chlorosis, mosaic	NA	Leaf deformation, blistering, necrosis, chlorosis, mosaic	+
	*Solanum lycopersicum* Roma	6/2	Leaf deformation, necrosis, chlorosis, mosaic	NA	Leaf deformation, blistering, necrosis, chlorosis, mosaic	+/-
	*Solanum melongena* Black beauty	8/0	Local lesions	+	None	-
	*Capsicum annuum* Soroksari	0/8	None	NA	None	-
	*Solanum tuberosum* Pentland	0/8	None	-	None	-
	*Solanum tuberosum* Igor	0/8	None	NA	None	-
	*Solanum tuberosum* Desiree	0/8	None	-	None	-
	*Nicotiana glutinosa*	8/0	Necrosis, mosaic	NA	Mosaic	+
	*Nicotiana tabacum* Samsun	8/0	Necrosis, mosaic	NA	Mosaic	+
	*Nicotiana benthamiana*	8/0	Leaf deformation	NA	Leaf deformatons dark coloration, chlorosis	+
	*Hyoscyamus niger*	8/0	Necrosis, mosaic	NA	Mosaic, blistering	+
	*Datura stramonium*	4/4	Wilting, yellowing, mosaic	NA	Wilting, yellowing, mosaic	+/-
	*Physalis floridana*	2/6	Leaf deformation, blistreing	NA	Leaf deformation, blistering, dwarf growth	+/-
Brassicaceae	*Brassica oleracea*	0/8	None	NA	None	-
	*Brassica rapa*	0/8	None	NA	None	-
Cucurbitaceae	*Cucumis melo*	0/8	None	NA	None	-
Amaranthaceae	*Chenopodium quinoa*	0/8	None	NA	None	-
	*Chenopodium amaranticolor*	0/8	None	NA	None	-
	*Amaranthus* sp.	0/8	None	NA	None	-

*NA, not applicable; +, positive RT-PCR result; -, negative RT-PCR result*.

Out of four isolates obtained from the virus collections, HMV-PV-76 was the only isolate present in a single infection. Thus, additionally we also checked the pathogenicity of the isolate HMV-PV-76 and compared it to the HMV-SI/L isolate by inoculating *N. benthamiana* and *S. lycopersicum* cv. Moneymaker using the same method as described in section “First Test on Sample HMV-SI/L.”

### Transmission Electron Microscopy (TEM)

**FIGURE 1:**
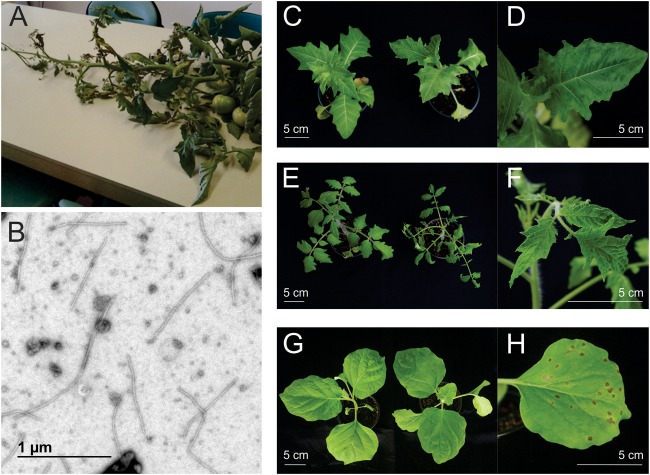
Disease symptoms on the original field-grown tomato sample and test plants infected for host range analysis with HMV-SI/L isolate. **(A)** Infected field-grown tomato plant with severe necrotic symptoms brought to laboratory for diagnostic investigation. **(B)** Viral particles of HMV-SI/L from mechanically inoculated *Nicotiana benthamiana* sample, visualized by transmission electron microscopy. **(C–H)** Disease symptoms caused by HMV-SI/L isolate on selected plant species included in the host range analysis. **(C)** Left: mock-inoculated *H. niger*, right: HMV-SI/L inoculated *H. niger*. **(D)** *H. niger* infected leaf showing blistering. **(E)** Left: mock-inoculated *S. lycopersicum* cv. Moneymaker, right: HMV-SI/L inoculated *S. lycopersicum* cv. Moneymaker showing leaf deformation. **(F)** S. *lycopersicum* cv. Moneymaker infected leaf showing blistering. **(G)** Left: mock-inoculated *S. melongena*, right: HMV-SI/L inoculated *S. melongena*. **(H)** *S. melongena* inoculated leaf showing necrotic lesions (local symptoms).

The original sample, selected test plants (see “First Test on Sample HMV-SI/L”) and leaves from *S. lycopersicum* and *N. benthamiana* (Figure 1B) infected with the HMV-SI/L isolate were examined using TEM. The sample (20 μl) was applied to Formvar-coated, carbon-stabilized copper grids and negatively stained using a 1% aqueous solution of uranyl acetate (SPI Supplies), followed by visualization using Philips CM 100 transmission electron microscope (FEI, Eindhoven, Netherlands). Images were captured using an ORIUS SC 200 CCD camera (Gatan Inc., Pleasanton, United States).

## Results

### Field-Grown Tomato Sample Contained HMV in a Mixed Viral Infection

A sample from field-grown tomato plant (*S. lycopersicum* L.) with severe necrotic symptoms (Figure 1A) was brought to the laboratory for a diagnostic investigation. The sample was analyzed for several different plant viruses and gave a positive result for PVM using ELISA. The presence of virus particles matching carlavirus morphology was confirmed by TEM. After the initial mechanical inoculation of nine different species of test plants, five of them (*N. benthamiana, N. glutinosa, C. quinoa, D. stramonium, C. annuum*) did not show any symptoms, the ELISA assay was negative for PVM and no virus particles were observed in those plant samples. Four of the test plants (*S. lycopersicum* cv. Moneymaker, *N. rustica, N. tabacum* cv. White Burley, *N. clevelandii*) were PVM positive by ELISA (the results were also confirmed by TEM). Since the disease symptoms observed on the original sample (Figure 1A) and on the tomato test plants (Supplementary Figure 1) were not typical for infection with PVM alone, the original plant sample was further analyzed. Total RNA was isolated from the original sample and sequenced using sRNA and rRNA depleted total RNA approaches. Henbane mosaic virus (HMV) was detected in both HTS data sets in mixed infection with southern tomato virus (STV) and with the previously detected PVM. The presence of HMV in the original sample and in the symptomatic test plants was confirmed using a RT-PCR assay designed in this study. The presence of STV was confirmed using RT-PCR (Sabanadzovic et al., 2009) only in the original plant sample and was not detected in the test plants, which was an expected outcome, since STV is not known to be mechanically transmissible (Sabanadzovic et al., 2009).

### HMV Was Mostly Present in Isolates From Viral Collections in Mixed Infections

**FIGURE 2:**
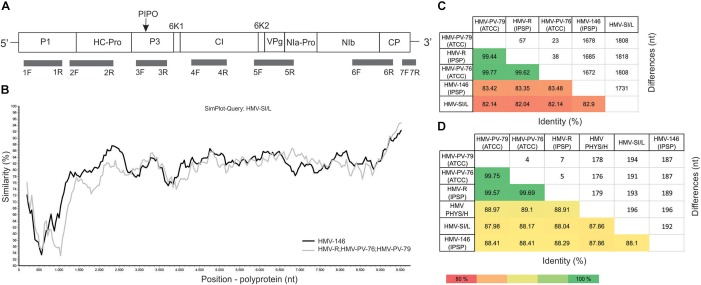
HMV genome representation, position of RT-PCR primers used in this study and pairwise comparisons of sequenced isolates. **(A)** Scheme of HMV genome. The positions and lengths of the protein products are indicated with corresponding box lengths. Below, the locations of all primers described in Supplementary Table 2 are marked. **(B)** The SimPlot graph showing the nucleotide similarity (%) across the HMV polyprotein between HMV-SI/L (query), HMV-146 (black line), and HMV-R, HMV-PV-76 and HMV-PV-79 (as a group, gray line). **(C)** Heatmap showing the results of pairwise comparisons between nucleotide sequences of whole genomes of HMV-SI/L, HMV-R, HMV-146, HMV-PV-76, and HMV-PV-79. Average nucleotide identities (%) are shown below the diagonal, number of different nucleotides are shown above the diagonal. **(D)** Heatmap showing the results of pairwise comparisons between partial genome sequence (1600 nt) of HMV-SI/L, HMV-R, HMV-146, HMV-PV-76, HMV-PV-79, and HMV-PHY/H (AM184113). Average nucleotide identities (%) are shown below the diagonal, number of different nucleotides are shown above the diagonal.

Before this study only a partial genome sequence of a single isolate of HMV was present in the public database, with no affiliated publication and little metadata (NCBI GenBank Acc. No. AM184113). The overlapping part of HMV-SI/L isolate sequenced in this study was 88% identical to this sequence, leading to a putative identification of the virus as HMV. To validate this putative identification, we obtained four other isolates designated as HMV from virus collections (HMV-R, HMV-146, HMV-PV-76, and HMV-PV-79) and performed sRNA sequencing to obtain their complete genome sequences. Using the sRNA virus detection pipeline (Pecman et al., 2017) we detected potyvirus sequences in all four samples. They had high sequence identities (Figure 2C) to the putative HMV isolate from tomato (HMV-SI/L). In all four samples HMV was detected and confirmed using the HMV-UNI RT-PCR assay. However, HTS analysis revealed that three out of four isolates contained sequences of other virus species, specifically: HMV-R and HMV-146 contained also *Potato aucuba mosaic virus* (*Potexvirus*, *Alphaflexiviridae*) and HMV-PV-79 contained *Potato virus Y* (*Potyvirus*, *Potyviridae*).

### Genome Characteristics of HMV

Complete consensus genome sequences were reconstructed for all five HMV isolates included in the study and deposited into NCBI GenBank (Supplementary Table 1). They had a genome organization typical for the genus *Potyvirus* with a single open reading frame (ORF) encoding a polyprotein of 3248–3249 amino acids and an estimated molecular weight of 367 kDa. The ORF encoded all typical potyviral protein products, with an unusually long P1 protein (476 amino acid residues) (Figure 2A). Additionally, one of the isolates (HMV-PV-76) contained an uncommon Nib-Pro/CP cleavage site (Q/V) which has previously been reported for an isolate of lettuce mosaic virus (Dinant et al., 1991) and was later found also in one isolate of potato virus Y (Blanco-Urgoiti et al., 1998) and several other potyviruses (Adams et al., 2005a). All HMV isolates had the PIPO frame shift protein within the P3 cistron and HC-Pro motifs involved in aphid transmission: RITC_51-54_ and PTK_309-311_. The detailed data about the polyprotein cleavage sites, molecular weights and lengths of products are presented in the Supplementary Table 3.

### Investigated HMV Isolates Cluster Into a Monophyletic Group and Contain High Within-Group Diversity

**FIGURE 3:**
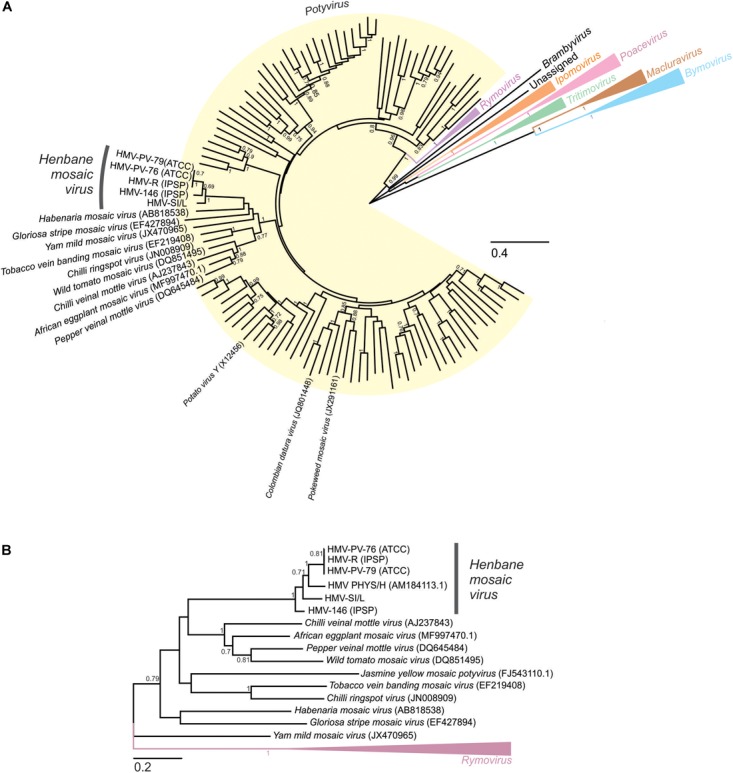
Maximum likelihood phylogenetic trees showing the relationship between *Henbane mosaic virus* and other member of *Potyviridae* family. Scale bars represent maximum-likelihood estimates of the number of substitution per site. **(A)** Phylogenetic tree based on the codon-aligned nucleotide sequences of the polyproteins of fully sequenced members of the family *Potyviridae*. Sequences were codon aligned using MUSCLE in MEGA. **(A)** Maximum Likelihood tree was constructed using the General Time Reversible (+G + I) substitution model and 100 bootstrap replicates; only bootstrap support values of 0.7 or above are shown for clarity reasons. The *Potyvirus, Rymovirus, Brambyvirus, Tritimovirus, Poacevirus, Ipomovirus, Macluravirus*, and *Bymovirus* genera are highlighted in yellow, purple, black, green, pink, orange, brown and blue, respectively, and labeled accordingly. **(B)** Maximum likelihood phylogenetic tree constructed from the alignment of partial (1600 nt) genome nucleotide sequences of the viral species clustering in the same group as HMV (see Figure 3A) and *Rymovirus* representatives used as an outgroup. Sequences were codon aligned using MUSCLE in MEGA. A Maximum Likelihood tree was then constructed using the General Time Reversible (+G + I) substitution model and 1000 bootstrap replicates; only bootstrap values of 0.7 or above are shown for clarity reasons.

In the phylogenetic analysis based on the alignment of the complete nucleotide polyprotein sequences of HMV isolates and other species from the *Potyviridae* family (Figure 3A), the HMV isolates were positioned within the *Potyvirus* genus. They represented a monophyletic group, which clustered within the subgroup of potyvirus species, together with: *Habenaria mosaic virus* (AB818538), *Gloriosa stripe mosaic virus* (EF427894), *Yam mild mosaic virus* (JX470965), *Tobacco vein banding mosaic virus* (EF219408), *Chili ringspot virus* (JN008909), *Wild tomato mosaic virus* (DQ851495), *Chili veinal mottle virus* (AJ237843), *African eggplant mosaic virus* (MF997470), and *Pepper veinal mottle virus* (DQ645484). Phylogenetic analysis based on the alignment of the smaller subset of partial genome sequences (Figure 3B) enabled us to also include the Hungarian isolate of HMV (HMV PHYS/H, AM184113) into the analysis, positioning it within the HMV cluster.

In both phylogenetic trees, three HMV isolates (HMV-R, HMV-PV-76, and HMV-PV-79) clustered together with high bootstrap support. HMV-146 and HMV-SI/L were significantly different from the HMV-R, HMV-PV-76, HMV-PV-79 group, but also from each other, thus they represented separate groups on the tree (Figure 3A). HMV PHYS/H was also different from all other isolates, not clustering with any of them on the tree (Figure 3B). To evaluate similarities and differences between HMV isolates in a greater detail, SimPlot analysis and pairwise comparisons of nucleotide sequences were performed. The pairwise comparisons was used to calculate the average percentage of identity and the number of differences in nucleotides (i) using whole genomes of all five HMV isolates (Figure 2C) and (ii) using partial genome sequence enabling the inclusion of the Hungarian isolate (AM184113) (Figure 2D). The highest percent of identity (above 99%) and thus the lowest number of differences were between HMV-R, HMV-PV-76, HMV-PV-79 isolates. The similarity of HMV-146 and HMV-SI/L to the above mentioned isolates and to each other was about 82% (Figure 2C). HMV PHYS/H identity to all five above mentioned HMV isolates was in each case around 88% (Figure 2D). Taken together, the results show that six HMV isolates sequenced to date represent four divergent phylogenetic groups, which may correspond to four distinct viral strains.

SimPlot analysis (Figure 2B) demonstrated the similarity between the three putative strain groups across the polyprotein sequence, specifically: between HMV-SI/L (used as query), HMV-R, HMV-PV-76, HMV-PV-79 isolates (grouped as one, since their similarity was above 99%) and HMV-146. A significant drop in similarity between the groups is observed in the P1 protein region; subsequent blastx (against NCBI *nr*) and blastn (against NCBI *nt*) similarity searches did not show significant similarity (i.e., identity > 40%) of HMV P1 product to any known sequence.

### HMV-SI/L Causes Symptomatic Infection of Several Solanaceous Species

Host range analysis for the HMV-SI/L isolate (detailed in Table 1) showed that eight of ten tested solanaceous species are hosts for HMV, which was confirmed by observation of disease symptoms and positive RT-PCR tests for HMV. For *H. niger, S. lycopersicum* (Figures 1C–F) and plants from the genus *Nicotiana* all or almost all (6–8) of the eight inoculated plants expressed disease symptoms 3 weeks after mechanical inoculation. Smaller number of *D. stramonium* (4/8) and *P. floridana* (2/8) plants expressed disease symptoms. Most severe disease symptoms were observed in the following two cases: in *D. stramonium*, where 4 of 8 plants died 6 weeks post inoculation and in *P. floridana*, where plants showed very strong dwarf growth. In *Solanum melongena*, the HMV did not move from the inoculated leaves and local lesions developed (Figures 1G,H) without developing systemic infection. This was confirmed by positive RT-PCR test results for inoculated leaves and negative RT-PCR test results for non-inoculated leaves, sampled three and four (data not shown) weeks post inoculation. Pooled samples of plants with no disease symptoms were RT-PCR negative and none of these plants showed disease symptoms not even 7 or 8 weeks after the inoculation.

Additional comparison of the pathogenicity between isolates HMV-SI/L and HMV-PV-76 (the latter is 99% identical to HMV-R on genome level) in *N. benthamiana* and *S. lycopersicum* cv. Moneymaker revealed more severe symptoms following infection with isolate HMV-PV-76. Three weeks after inoculation, plants of *N. benthamiana* infected with HMV-PV-76 showed more severe symptoms (curling, blistering, yellowing) than plants infected with HMV-SI/L, and died 4 weeks after inoculation. Similarly, in the case of *S. lycopersicum* cv. Moneymaker, the symptoms induced by HMV-PV-76 were more severe (blistering, curling, dwarf growth, parsley leaves), in comparison to HMV-SI/L (Supplementary Figure 2).

## Discussion

High-throughput sequencing has in the recent decade facilitated discovery of many new viruses and viral strains and largely improved the possibilities for broad range virus detection and screening. The increased rate of virus discovery, also brings new challenges, especially considering the biological characterization of the newly detected viral sequences. The latter is crucial to establish their importance, i.e., their impact on agriculture and trade (Massart et al., 2017) or their natural ecosystems. Since this process comprises laborious classical virology techniques, in many cases, the biological characterization of rapidly discovered new viral species is lagging behind their discovery, often simply due to time constrains.

However, for henbane mosaic virus described here, the perspective was reversed; very limited genomic information was available for a virus first described in 1932 (Hamilton, 1932) and subsequently characterized biologically. Here, firstly, HTS was used as a reliable generic detection technique for identifying the presence of known and unknown viruses in field samples, which resulted in the detection of HMV for the first time in tomato and the first time in any plant in Slovenia. Since the virus was present in a mixed infection, and little genomic information was available at the time, it was overlooked by routinely used diagnostic methods. Secondly, HTS enabled us to rapidly generate complete genomic sequences of several isolates of the HMV, which was followed by their genome annotation and phylogenetic analyses.

Although HMV detection and characterization was reported 86 years ago, the correct taxonomic determination of the virus detected in field-grown tomato was uncertain due to the lack of genomic information in sequence databases. To overcome this problem, we obtained, sequenced and analyzed four other HMV isolates from different virus collections using the sRNA sequencing approach. This enabled us, first, to characterize the HMV genome, which has typical potyvirus organization and contains an unusually long P1 protein, known for its great variability within the genus (Adams et al., 2005b). Consequently, HMV has one of the longest genomes in the genus, which is in agreement (Cui et al., 2014) with previous observations of longer virus particles (Lovisolo and Bartels, 1970). Secondly, the genomic information obtained enabled us to perform phylogenetic analyses and taxonomically classify the HMV isolates within the *Potyvirus* genus. Interestingly, HMV is placed within the chili veinal mottle virus cluster and is not closely related to any of the virus species (*Potato virus Y*, *Colombian datura virus*, or *Pokeweed mosaic virus*), for which a distant serological relationship with HMV has been previously speculated (Lovisolo and Bartels, 1970; Govier and Plumb, 1972; Figure 3A). Further, pairwise comparison of HMV isolates genomic sequences revealed that three differently named isolates (HMV-PV-76, HMV-PV-79, and HMV-R) were very similar (99% identity), while the other two (HMV-SI/L and HMV-146) form distinct phylogenetic clusters (Figures 2, 3). Sequence variability between HMV-SI/L and HMV-PV-76 was reflected also in a different pathogenicity on *N. benthamiana* and *S. lycopersicum* cv. Moneymaker (Supplementary Figure 2). According to the sequence similarities within other potyvirus species (Adams et al., 2005b) these three units might belong to 3 different strain groups within the same species. The partially sequenced HMV-PHYS/H isolate detected in Hungary, might belong to the 4th group. The observed sequence diversity among the low number of analyzed HMV isolates is surprisingly high. This could be explained by several scenarios. They were isolated from different countries, different host plants and put into the collections at different decades during the last 80 years (Supplementary Table 1). To estimate the current diversity among different HMV isolates in nature, additional studies, including several samples collected from the natural environment, from different host plants and from a broader geographic range, should be completed.

Previously, the relationships between different HMV isolates were examined mainly based on their biological properties, e.g., host range and symptomatology. Although sequenced isolates described in this paper are probably not identical as the isolates described in previously published biological studies, the comparison between them, considering the host range and symptomatology, could give us an additional insight into their diversity. Susceptible hosts of a HMV-SI/L isolate with pronounced disease symptoms were closely related plant species, e.g., *H. niger*, *Nicotiana* spp. and *S. lycopersicum* (different varieties). Similar results were obtained by Lovisolo and Bartels (1970) for HMV-R and HMV-A. In our study, HMV-SI/L caused a hypersensitive reaction (showing local necrotic lesion and no systemic infection) in *S. melongena*. On the other hand, HMV-R showed no disease symptoms on *S. melongena* (Hamilton, 1932), thus it could be used as a test plant to discriminate between HMV-SI/L from HMV-R. HMV-SI/L did not infect *C. annuum*; however, taking different reports into account, there are contradicting results whether HMV-R can infect *C. annuum* (Lovisolo and Bartels, 1970) or not (Salamon, 2018). HMV-SI/L did not infect *Chenopodium amaranticolor* or *C. quinoa* in our study. However, HMV-R and HMV-A isolates induced disease symptoms on *C. amaranticolor* (Lovisolo and Bartels, 1970) and, in another study, HMV-R did not induce symptoms on *C. quinoa* (Salamon, 2018).

Nevertheless, all the previously reported host range analysis should be interpreted with caution. The composition of those inoculums was not confirmed with HTS, so they could have contained mixed infections of viral species, which could explain some of the contradictory results. Indeed, HTS analysis done here on HMV isolates obtained from virus collections showed that three out of four HMV isolates were present in mixed infections. The host range analysis done in our study is the first one in conducted where single infection of the inoculum was confirmed. Furthermore, when comparing the observed symptoms of mixed infection from the field tomato sample (Figure 1A, severe necrotic symptoms) with symptoms of HMV-SI/L single infection of tomato plant in the greenhouse (Figures 1E,F and Table 1), the symptomatology is different, possibly due to the fact that the virus was present in mixed infection in the originally sampled tomato plant. We demonstrated that HTS can help improving complex plant disease etiology investigations, as a fast screening tool, implemented prior to biological characterization studies. It helps to assess the possibility of mixed infections. In addition, HTS can be used as an effective tool for screening and revision of the already established virus collections, to obtain more accurate status of the deposited isolates.

The study of HMV presented in this paper demonstrated the benefit of using HTS for characterization of known viral species with limited or absent genomic information. The *Potyvirus* genus (*Potyviridae* family) is the largest genus of plant viruses, including 168 virus species, which cause diseases in a wide range of plant species. Among them, according to the latest ICTV report, 114 have complete genome sequences available, for 48 there are just partial genomic sequences available, and for 6 of them there is no sequence data in public repositories (ICTV, 2017); the latter two categories likely representing a similar scenario as that described in this study. It is likely that these viruses are currently not a major concern regarding agricultural production and trade and thus less research effort has been focused on them. However, such viruses can become emergent problems in the future, since they constantly evolve and adapt to new environments and can switch hosts (Geoghegan et al., 2017). As a result, they might become emerging problems if cultivated plants become natural hosts for some of them in the future. We demonstrated that HTS can be used to characterize full genomes of such viruses. This improves information in the databases by adding missing sequences, enabling more rapid development of diagnostic assays, leading to quicker response times for emerging disease problems.

## Author Contributions

AP, MR, DK, and NM designed the experiments. PP discovered new type of disease symptoms on tomato, started further investigation, and provided the field tomato sample infected with HMV-SI/L. NM performed first screening analysis on the field tomato sample. AP performed laboratory part of the experiments and analyzed the data with the assistance of IA and DK. MŽ recorded TEM photos. AP wrote the draft of the manuscript. All authors significantly contributed with reviewing and editing the manuscript.

## Conflict of Interest Statement

IA was employed by company Fera Science Ltd., Sand Hutton, York, United Kingdom and PP was employed by company KZ Agraria Koper z.o.o., Koper, Slovenia. The remaining authors declare that the research was conducted in the absence of any commercial or financial relationships that could be construed as a potential conflict of interest.

## Footnotes

1 https://www.ncbi.nlm.nih.gov/Sequin/index.html

2 https://www.sciencegateway.org/tools/proteinmw.htm

3 https://talk.ictvonline.org/ictv-reports/ictv_online_report/positive-sense-rna-viruses/w/potyviridae/565/resources-potyviridae
