# Supplementary figures and images for: High-Throughput Sequencing Facilitates Characterization of a “Forgotten” Plant Virus: The Case of a Henbane Mosaic Virus Infecting Tomato
Source: Front Microbiol. 2018 Nov 19;9:2739. doi: 10.3389/fmicb.2018.02739 (PMC6254090; doi:10.3389/fmicb.2018.02739)

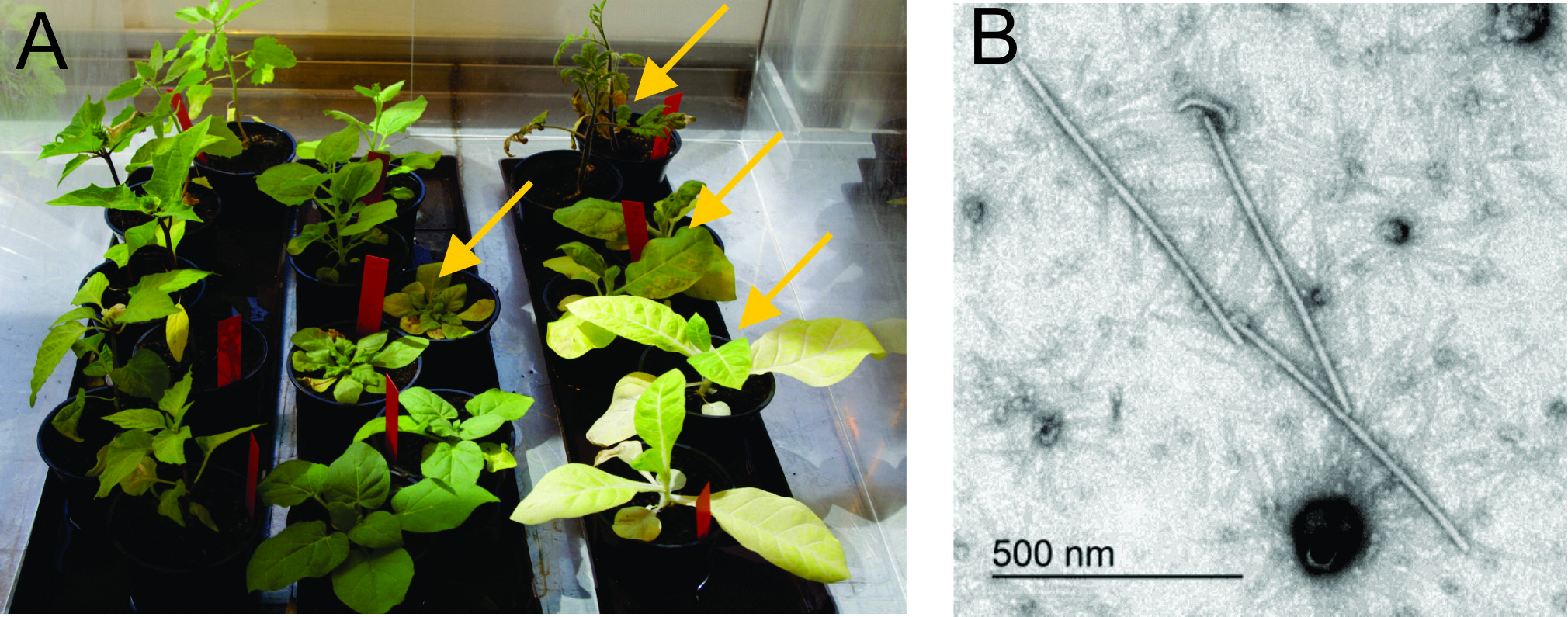

Supplement: Supplementary file 1 [file Image_1.JPEG]

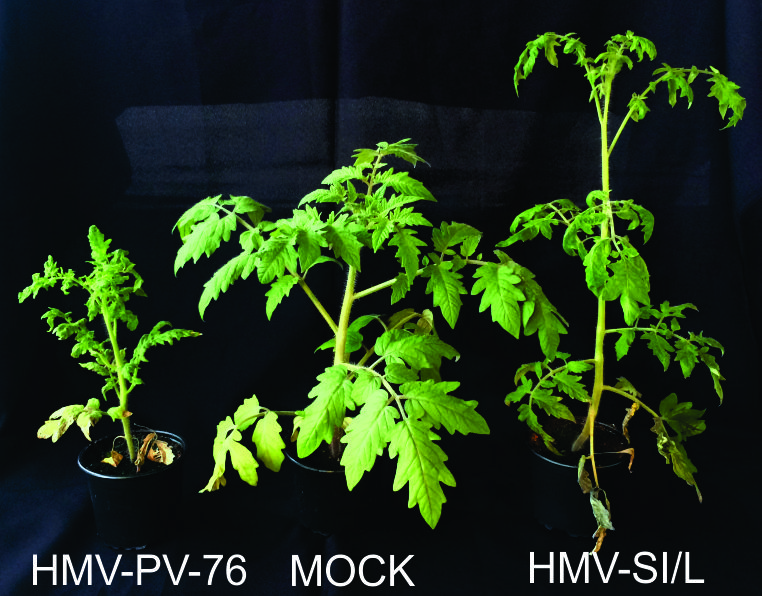

Supplement: Supplementary file 2 [file Image_2.JPEG]
