# Supplementary material for: High-Throughput Sequencing Facilitates Characterization of a “Forgotten” Plant Virus: The Case of a Henbane Mosaic Virus Infecting Tomato
Source: Front Microbiol. 2018 Nov 19;9:2739. doi: 10.3389/fmicb.2018.02739 (PMC6254090; doi:10.3389/fmicb.2018.02739)
Supplement: Supplementary file 3 [file Data_Sheet_1.docx]

Supplementary Material

High-throughput sequencing facilitates the characterisation of the “forgotten” plant virus: A case of the novel henbane mosaic virus infecting tomato

**Anja Pecman^1,2^*, Denis Kutnjak^1^, Nataša Mehle^1^, Magda Tušek Žnidarič^1^, Ion Gutiérrez-Aguirre^1^, Patricija Pirnat^3^, Ian Adams^4^, Neil Boonham^5^, Maja Ravnikar^1,6*^**

***Correspondence:**Anja Pecman
[anja.pecman@nib.si](mailto:anja.pecman@nib.si)

Maja Ravnikar

[maja.ravnikar@nib.si](mailto:denis.kutnjak@nib.si)

# Supplementary Figures and Tables

## Supplementary Table

Supplementary Table 1: Detailed information about HMV isolates described in the study.

| Isolate name in manuscript | Isolate name | Original host plant; laboratory host plant in which the virus was maintained | Virus collection (organization) | Date (DD/MM/YYYY), place of sampling and collector (if known) | Detected virus species | NCBI GenBank accession number for HMV | NCBI SRA accession number (sRNA/rRNA depleted totRNA) |
| --- | --- | --- | --- | --- | --- | --- | --- |
| HMV-SI/L | HMV-SI/L | *Solanum lycopersicum*; maintained in *Solanum lycopersicum* | National Institute of Biology | 24/06/2015 Ankaran, Slovenia, collected by Patricija Pirnat | *Henbane mosaic virus, Potato virus M, Southern tomato virus* | MH779472 | SRR7734364/ SRR7734393 |
| HMV-R | HMV-R (Rothamsted) | unknown; maintained in *Nicotiana tabacum* | Institute for Sustainable Plant Protection | Originating from Rothamsted, 01/01/1963 - was collected by Dr. Osvaldo Lovisolo | *Henbane mosaic virus, Potato aucuba mosaic virus* | MH779474 | SRR7734366/ NA |
| HMV-146 | HMV-146 | *Datura inermis*; maintained in *Datura stramonium* | Institute for Sustainable Plant Protection | 19/8/1964, Torino (Botanic Garden), collected by Dr Osvaldo Lovisolo | *Henbane mosaic virus, Potato aucuba mosaic virus* | MH779473 | SRR7734365/ NA |
| HMV-PV-76 | California | *Hyoscamus niger*, Califormia; maintained in *N. tabacum* cv. Samsun | American Type Culture Collection (ATCC) | See ATCC:  https://www.lgcstandards-atcc.org/products/all/PV-76 | *Henbane mosaic virus* | MH779475 | SRR7734367 /NA |
| HMV-PV-79 | Watson's Isolate A | *Hyoscamus niger*, England; maintained in *N. tabacum* cv. Samsun | American Type Culture Collection (ATCC) | See ATCC:  https://www.lgcstandards-atcc.org/products/all/PV-79 | *Henbane mosaic virus, Potato virus Y* | MH779476 | SRR7734368/ NA |

## NA – not applicable

Supplementary Table 2: List of primer pairs used for PCR amplification.

| Pair number | Primer name | Primer sequence 5ˈ-3ˈ | Primer position at HMV-SI/L sequences consensus |
| --- | --- | --- | --- |
| 1 | P1start-F  P1-R | CTTACTCTTCCTCCCCCA  ATTGTTCTGTTGTTCCTCC | 331-348  1268-1286 |
| 2 | P1end-F  HCpro-R | GCGGTTTTATCTTTCCAGT  TGATTCTTTGTGGGTGTCTT | 1469-1487  2536-2555 |
| 3 | P3-F  P3-R | TTCTCACCAAGCATTCTC  CATCTTCTCATCACCATCA | 3115-3132  3867-3885 |
| 4 | Cl-F  Cl-R | AGAGTTGAGTAAGAGGGGG  TAAAACACAGAAATGCCGCC | 4488-4506  5349-5368 |
| 5 | Clend-F  NIaPro-R | ACATCAAGTAGAGAGCGG  GGTTCGGAGTGTTAGTGT | 6042-6059  7012-7029 |
| 6 | HMV-NIb-F  HMV-CP-R | GTCAAGAAGTTCAAAGGG  TACACCACACCATCAATC | 8479-8496  9473-9490 |
| 7 | HMV-UNI-F  HMV-UNI-R | TTAGCCCGATATGCTTTC  CTATCTTCCACTTCAGGT | 9715-9732  10043-10060 |

**Supplementary Table 3:** Detailed description of annotated polyproteins for HMV isolates: HMV-SI/L, HMV-146, HMV-R, HMV-PV-76 and HMV-PV-79. Information about virus genome and its polyprotein length (nt) are given for each isolate. The corresponding number of amino acid residues, molecular weight (kilo dalton) and cleavage position for each protein are listed in separate lines. The PIPO position is indicated at the bottom of the table.

|  | | HMV-SI/L | HMV-146 | HMV-R | HMV-PV-76 | HMV-PV-79 |
| --- | --- | --- | --- | --- | --- | --- |
| Virus length (nt)/  Virus polyprotein length (nt) | | 10127/  9747 | 10125/  9747 | 10140/  9750 | 10140/  9750 | 10140/  9750 |
| POLYPROTEIN | Residues No. | 3248  368.04 | 3248  367.65 | 3249  367.19 | 3249  367.26 | 3249  367.27 |
|  | Mw (kDa) |  |  |  |  |  |
| P1 | Residues No. | 476  54.5  MQYY_476_-S | 476  54.3  MRYY_476_-S | 477  53.82  MRYY_477_-S | 477  53.78  MRYY_477_-S | 477  53.78  MRYY_477_-S |
|  | Mw (kDa) |  |  |  |  |  |
|  | Cleavage position |  |  |  |  |  |
| HC-Pro | Residues No. | 457  51.54  YQVG_933_-G | 457  51.6  YQVG_933_-G | 457  51.42  YQVG_934_-G | 457  51.4  YQVG_934_-G | 457  51.4  YQVG_934_-G |
|  | Mw (kDa) |  |  |  |  |  |
|  | Cleavage position |  |  |  |  |  |
| P3 | Residues No. | 348  40.14  VKHQ_1281_-G | 348  40.05  VKHQ_1281_-A | 348  40.14  VKHQ_1282_-A | 348  40.15 VKHQ_1282_-A | 348  40.15  VKHQ_1282_-A |
|  | Mw (kDa) |  |  |  |  |  |
|  | Cleavage position |  |  |  |  |  |
| 6K1 | Residues No. | 54  6.09  VTHQ_1335_-S | 54  6.14  VTHQ_1335_-S | 54  6.12  VAHQ_1336_-S | 54  6.12  VAHQ_1336_-S | 54  6.12  VAHQ_1336_-S |
|  | Mw (kDa) |  |  |  |  |  |
|  | Cleavage position |  |  |  |  |  |
| CI | Residues No. | 645  71.87  VMHQ_1980_-S | 645  71.76  VMHQ_1980_-S | 645  71.83  VMHQ_1981_-S | 645  71.89  VMHQ_1981_-S | 645  71.92  VMHQ_1981_-S |
|  | Mw (kDa) |  |  |  |  |  |
|  | Cleavage position |  |  |  |  |  |
| 6K2 | Residues No. | 53  5.91  VAHQ_2033_-G | 53  5.86  VAHQ_2033_-G | 53  5.9  VAHQ_2034_-G | 53  5.9  VAHQ_2034_-G | 53  5.9  VAHQ_2034_-G |
|  | Mw (kDa) |  |  |  |  |  |
|  | Cleavage position |  |  |  |  |  |
| VPg | Residues No. | 163  18.49  PEQE_2196_-G | 163  18.49  PEYE_2196_-G | 163  18.57  PEYE_2197_-G | 163  18.57  PEYE_2197_-G | 163  15.57  PEYE_2197_-G |
|  | Mw (kDa) |  |  |  |  |  |
|  | Cleavage position |  |  |  |  |  |
| NIa-Pro | Residues No. | 267  30.36  VVEQ_2463_-A | 267  30.28  VTEQ_2463_-A | 267  30.29  VTEQ_2464_-A | 267  30.29  VTEQ_2464_-A | 267  30.29  VTEQ_2464_-A |
|  | Mw (kDa) |  |  |  |  |  |
|  | Cleavage position |  |  |  |  |  |
| NIb | Residues No. | 516  59.06  VYHQ_2979_-G | 516  59.04  VYHQ_2979_-A | 516  58.93  VYHQ_2980_-A | 516  58.93  VYHQ_2980_-V | 516  58.93  VYHQ_2980_-A |
|  | Mw (kDa) |  |  |  |  |  |
|  | Cleavage position |  |  |  |  |  |
| CP | Residues No. | 269  30.24 | 269  30.31 | 269  30.33 | 269  30.39 | 269  30.37 |
|  | Mw (kDa) |  |  |  |  |  |

PIPO: at polyprotein sequence position: 3267 nt – 3473 nt for HMV-R, HMV-PV-76 and HMV-PV-79 isolates and 3264 nt – 3470 nt for: HMV-SI/L and HMV-146 isolates.

## Supplementary Figure

**Supplementary figure 1**: Diagnostic screening testing. A) Mechanically inoculated test plants with developed symptoms (yellow arrows indicate symptomatic plants of *Solanum lycopersicum* cv. Moneymaker, *Nicotiana rustica, Nicotiana tabacum* cv. White Burley, *Nicotiana clevelandii*). B) Observed viral particles (using TEM) in mechanically inoculated test plants.

**Supplementary figure 2:** Disease symptoms on tomato plants (*Solanum lycopersicum* cv. Moneymaker) three weeks after inoculation with: HMV-PV-76 (left), MOCK (middle) and HMV-SI/L (right). Tomato plants were mechanically inoculated as explained in section 2.2.
